# Identification of tumour immune microenvironment-related alternative splicing events for the prognostication of pancreatic adenocarcinoma

**DOI:** 10.1186/s12885-021-08962-7

**Published:** 2021-11-12

**Authors:** Bo Chen, Tuo Deng, Liming Deng, Haitao Yu, Bangjie He, Kaiyu Chen, Chongming Zheng, Daojie Wang, Yi Wang, Gang Chen

**Affiliations:** 1 Key Laboratory of Diagnosis and Treatment of Severe Hepato-Pancreatic Diseases of Zhejiang Province, The First Affiliated Hospital of Wenzhou Medical University, Wenzhou, China; 2 Department of Hepatobiliary Surgery, The First Affiliated Hospital of Wenzhou Medical University, Wenzhou, China; 3 Division of Preventive Medicine, School of Public Health and Management, Wenzhou Medical University, Wenzhou, China

**Keywords:** Pancreatic adenocarcinoma, Alternative splicing, Tumour immune microenvironment, Prognosis, Immunotherapy

## Abstract

**Purpose:**

Pancreatic adenocarcinoma (PAAD) is characterized by low antitumour immune cell infiltration in an immunosuppressive microenvironment. This study aimed to systematically explore the impact on prognostic alternative splicing events (ASs) of tumour immune microenvironment (TIME) in PAAD.

**Methods:**

The ESTIMATE algorithm was implemented to compute the stromal/immune-related scores of each PAAD patient, followed by Kaplan–Meier (KM) survival analysis of patients with different scores grouped by X-tile software. TIME-related differentially expressed ASs (DEASs) were determined and evaluated through functional annotation analysis. In addition, Cox analyses were implemented to construct a TIME-related signature and an AS clinical nomogram. Moreover, comprehensive analyses, including gene set enrichment analysis (GSEA), immune infiltration, immune checkpoint gene expression, and tumour mutation were performed between the two risk groups to understand the potential mechanisms. Finally, Cytoscape was implemented to illuminate the AS-splicing factor (SF) regulatory network.

**Results:**

A total of 437 TIME-related DEASs significantly related to PAAD tumorigenesis and the formation of the TIME were identified. Additionally, a robust TIME-related prognostic signature based on seven DEASs was generated, and an AS clinical nomogram combining the signature and four clinical predictors also exhibited prominent discrimination by ROC (0.762 ~ 0.804) and calibration curves. More importantly, the fractions of CD8 T cells, regulatory T cells and activated memory CD4 T cells were lower, and the expression of four immune checkpoints—PD-L1, CD47, CD276, and PVR—was obviously higher in high-risk patients. Finally, functional analysis and tumour mutations revealed that aberrant immune signatures and activated carcinogenic pathways in high-risk patients may be the cause of the poor prognosis.

**Conclusion:**

We extracted a list of DEASs associated with the TIME through the ESTIMATE algorithm and constructed a prognostic signature on the basis of seven DEASs to predict the prognosis of PAAD patients, which may guide advanced decision-making for personalized precision intervention.

**Supplementary Information:**

The online version contains supplementary material available at 10.1186/s12885-021-08962-7.

## Background

Pancreatic adenocarcinoma (PAAD) is a gastrointestinal cancer with a bleak prognosis, accounting for almost as many deaths (466000) as cases (496000) among 185 countries in 2020 [1, 2]. As a silent tumour, only approximately 20% of PAAD patients with a resectable tumour can be diagnosed at an early stage due to the absence of typical clinical manifestations and sensitive screening regimens [3], resulting in a poor five-year overall survival rate of 5–8% [2, 4, 5]. Once PAAD patients develop metastatic disease, the median overall survival decreases to 6 months even with state-of-the-art treatment [6]. Thus, the development of screening strategies and therapeutic options remains an unmet need. Although the advent of next-generation sequencing technologies has enabled characterization of the molecular characteristics of PAAD [7], comprehensive analyses of the tumour immune microenvironment (TIME) to improve our understanding of tumour heterogeneity and to explore novel biomarkers for early diagnosis, prognostication, and targeted therapies have been insufficient [8].

Although considerable progress have been achieved in studies on PAAD genomic and immune landscapes and have recently fostered the exploitation of targeted therapies, they have only benefited a small number of patients [9]. According to the results of some clinical trials, pathway-specific targeted therapies have failed to provide clinically significant benefits for PAAD patients [6]. In addition, combining chemotherapy with immune checkpoint blockade therapy has been very successful in breast, lung, and gastric cancers [10, 11] but not in PAAD. Among the most immune-resistant cancers, dual checkpoint blockade targeting T cell inhibition, including CTLA-4 and PD-L1, also showed unsatisfactory results [12]. Although the reasons are not entirely clear, diverse and complex TIMEs containing not only PAAD cells but also immune cells, stromal cells, and bone marrow-derived cells may be responsible for the poor efficacy of targeted therapies [13]. More importantly, PAAD shows an immunologically “cold” TIME characterized by typical myeloid cell infiltration without CD8+ T cells [14], and eliminating immunosuppressive cells and elements within the TIME may resolve this conundrum [15]. Therefore, a compressive understanding of TIME complexity and heterogeneity, as well as biological interactions between PAAD cells and TIME components, may help to elucidate chemoresistance and explore novel diagnostic and therapeutic targets.

Previous studies have sought to delineate the special correlation between alternative splicing events (ASs) and the formation of the TIME of cancer cells [16]. ASs are some of the most important forms of mRNA processing at the posttranscriptional level, and approximately 94% of human genes are modified by ASs [17, 18]. Abnormal forms of ASs may cause structural and functional variation at the protein level, which drives a variety of malignant phenotypes, including those related to the angiogenesis, invasiveness, and chemoresistance of tumours [19, 20]. More importantly, numerous ASs have recently been determined to be neoantigens suitable for immunotherapy [21]. In addition, ASs can also regulate immunocytolytic mechanisms and affect the level of immune cell infiltration [22]. Although Lu et al. and Rong et al. systematically analysed prognostic AS events and their regulatory mechanisms in PAAD, neither study started from the perspective of the TIME. Therefore, we further investigated the underlying regulatory mechanisms between ASs, the TIME and prognosis in PAAD.

In this research, we utilized the ESTIMATE algorithm to evaluate the abundance of stromal and immune cells in PAAD patients by calculating corresponding scores and generated Kaplan–Meier curves to determine prognostic significance among patients with different stromal/immune scores. According to the differentially expressed ASs (DEASs) identified from the perspective of the TIME, we developed and validated a prognostic signature and an AS clinical nomogram. Moreover, comprehensive analyses were performed with respect to functional annotation, tumour mutation, immune infiltration, immune checkpoint gene expression, and AS-splicing factor (SF) networks were generated to identify the molecular mechanisms underlying the TIME and immunotherapy.

## Method and materials

### Data acquisition and preprocessing

RNA sequencing data of PAAD patients were obtained from the TCGA database (https://tcgadata.nci.nih.gov/tcga/), and corresponding clinical characteristics were extracted from the cBioPortal database. A total of 177 PAAD patients with complete RNA sequencing data and available survival information were enrolled in this study. Information on seven types of ASs was acquired from the TCGA SpliceSeq database [23], and the percent spliced in (PSI) value was applied to quantify them. To ensure the rigor of the study, only ASs with average PSI values larger than 0.05 were selected. Finally, 174 PAAD patients with appropriate AS data were included.

### Exploring the prognostic value of the stromal/immune scores of PAAD patients

Tumour cells promote various biological behaviour changes through direct or indirect interactions with stromal cells and immune cells, including inhibiting apoptosis, proliferation, angiogenesis, and immune tolerance [24]. To determine the proportions of stromal and immune cells in PAAD patients, stromal/immune scores were computed from the RNA sequencing data using the ESTIMATE algorithm. Moreover, we further investigated the association between stromal/immune scores and the prognosis of PAAD patients by classifying patients into high/low-stromal/immune score groups using X-tile software, which was utilized to infer the first-rank cut-off values of continuous variables for survival analysis. Subsequently, Kaplan–Meier (K-M) survival analyses were performed to assess the clinical significance of stromal/immune scores.

### Profiling differentially expressed ASs (DEASs) according to stromal/immune scores

To identify tumour-related ASs from the perspective of the TIME, differential analyses were performed based on the PSI values of ASs between PAAD patients in the low-stromal and high-stromal score groups and low-immune and high-immune score groups with the “limma” package. Considering that the PSI values of ASs, which ranged from 0 to 1, were relatively small, expression differences were assessed according to the relaxed standard of log2-fold change (log2FC). Only ASs with a false discovery rate (FDR) < 0.05 and |log2 FC| > 0.5 were determined to be differentially expressed ASs (DEASs). As the DEASs were identified between the different stromal/immune score groups, including upregulated ASs and downregulated ASs, we generated two Venn plots to screen valuable TIME-related DEASs that were upregulated or downregulated in the high-stromal and high-immune score groups. Accordingly, Upset plots were utilized to illustrate the interactive sets between the seven types of DEASs, as well as ASs before selection, including Alternate acceptor site (AA), Exon skip (ES), Retained intron (RI), Alternate terminator (AT), Mutually exclusive exon (ME), Alternative donor site (AD), and Alternate promoter (AP) [25] **(**Fig. 2C**)**.

### Functional annotation analysis

To further investigate the underlying mechanisms, the “Metascape” website tool was used (http://metascape.org/) for functional annotation analysis, which involved Gene Ontology (GO) and Kyoto Encyclopedia of Genes and Genomes (KEGG). Results with an adjusted *P* value < 0.05 were identified, and the top 20 important terms in both GO and KEGG analyses were visualized.

### Establishment of the TIME-related signature on the basis of DEASs

To illustrate OS-related DEASs in PAAD patients, we utilized univariate Cox regression analysis to mine DEASs with clinical significance. For the identified OS-related DEAS, we implemented LASSO regression analysis to select the most appropriate candidates with the “glmnet” package [26]. Finally, based on the optimal OS-related DEASs in the LASSO analysis, we established a TIME-related prognostic signature using the multivariate Cox proportional risk model. The risk score of individual PAAD patients was calculated using the following equation:

$$ riskScore=\sum \limits_{i=1}^n{\beta}_i\ast PSI\kern0.5em of\kern0.5em {DEAS}_i $$

Here, “ *β*_*i*_ ” is the regression coefficient of *DEAS*_*i*_, and *PSI of DEAS*_*i*_ is the PSI value of *DEAS*_*i*_.

To assess the performance of the signature, the median risk score was considered to be a uniform cut-off threshold to categorize the PAAD patients into two risk groups (high risk vs. low risk). Then, we generated K-M survival curves to graphically demonstrate OS between the two groups. Furthermore, time-dependent receiver operating characteristic (ROC) curves for predicting PAAD patients’ clinical outcomes at 1–3 years were employed to verify the performance of the signature.

### Independence test of the TIME-related signature and nomogram construction

PAAD patients with complete clinical predictors were enrolled to evaluate the independence of the TIME-related signature, which included sex, age, tumour size, tumour grade, margin status, radiotherapy, N stage, T stage, and AJCC stage. For this purpose, we implemented univariate and multivariate Cox regression analyses to mine independent predictors. The TIME-related signature and clinical characteristics with *P* < 0.05 in the multivariate analysis were incorporated into nomogram establishment with the “rms” package. Then, time-dependent ROC and calibration curves at 1–3 years were generated to visualize the discrimination of the AS clinical nomogram.

### Splicing correlation network construction

Splicing factors (SFs), which play a vital role in regulating ASs, were obtained from the SpliceAid2 database [27]. In total, data on 385 SF expression levels were extracted from the RNA sequencing of PAAD patients. Then, Pearson correlation analysis was implemented to identify the underlying regulatory mechanisms between OS-related DEASs and SFs. To develop a rigorous regulatory network, we set strictly restricted conditions of R > 0.8 and *P* < 0.0001 to denote a statistically significant relationship. In addition, to better illustrate the relationships between SFs and OS-related DEASs, the AS-SF network was constructed by Cytoscape [28].

### Evaluation of tumour mutation, immune infiltration, and immune checkpoint gene expression between the low-risk and high-risk groups

Two waterfall plots were generated to demonstrate genetic mutations in the low-risk and high-risk groups with the “maftools” packages. Moreover, to understand the immune characteristics between different risk groups, we utilized the “CIBERSORT” package to determine the fractions of 22 infiltrating immune cell types, and only PAAD patients with significant results (*P* < 0.05) were identified. Meanwhile, the expression levels of 15 immune checkpoint genes considered targets for cancer immunotherapy were extracted from the RNA-sequencing data. Then, Wilcoxon analysis was employed to compare the infiltration levels of the 22 immune cell types and the expression levels of the 15 immune checkpoint genes between different risk groups. In addition, significantly differentially expressed cells/genes were included in correlation analyses with riskScores for deeper exploration.

### Statistical analysis

All statistical analyses were implemented in R (version 4.0.2), and a *P* value < 0.05 (two-sided) was regarded as statistically significant with the exception of the selection of rigorous DEAS-related SPs (*P* < 0.0001).

## Results

### Stromal/immune scores are associated with the prognosis of PAAD patients

**Fig. 1:**
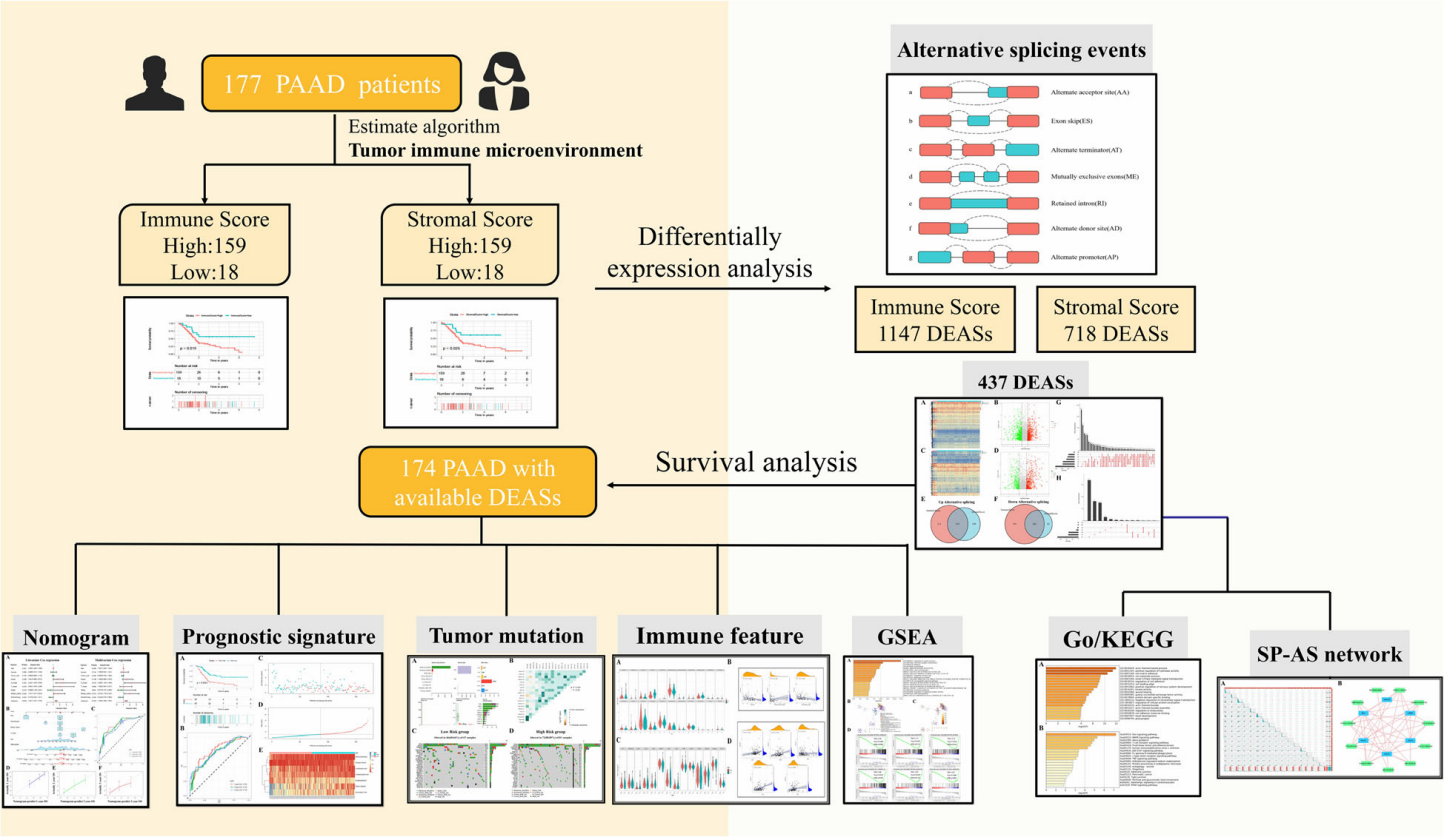
The flow chart of this study

**Fig. 2:**
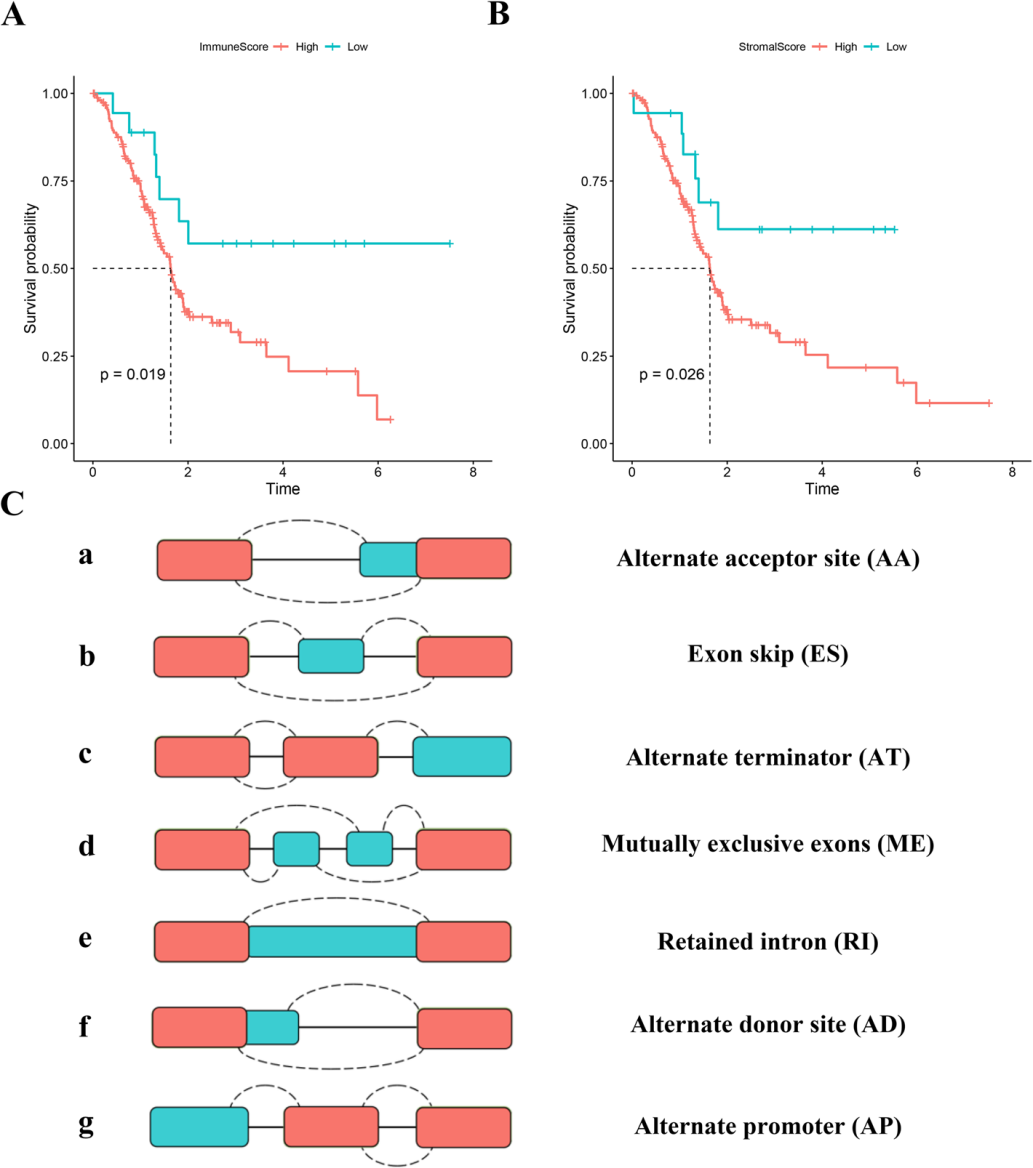
Stromal/immune scores are associated with the prognosis of PAAD patients. **A** Kaplan–Meier survival curves of overall survival (OS) for PAAD patients with high/low immune scores. **B** Kaplan–Meier survival curves of OS for PAAD patients with high/low stromal scores. **C** A schematic diagram displaying the seven types of alternative splicing events (ASs)

The flow chart of this study is shown in Fig. 1. In total, 177 PAAD patients with complete survival information and transcriptome data were enrolled, including 145 (81.92%) with pancreatic ductal adenocarcinoma (PDAC), 4 (2.26%) with pancreatic colloid carcinoma, 1 with pancreatic undifferentiated carcinoma, and 27 (15.26%) with other PAAD types, and their characteristics and clinical data are shown in Table 1. Overall, the stromal score of the cohort ranged from − 1463.77 to 1929.31, and the immune score was distributed between − 1026.21 and 2944.98. According to the X-tile (Supplementary Fig. 1), we divided patients into high/low-stromal score groups (cut-off value: − 318.43) and high/low-immune score groups (cut-off value: 188.30). Then, K-M survival curves implied that patients with lower stromal scores (*n* = 18) presented a favourable survival time compared to patients with higher stromal scores (*n* = 159) (*P* = 0.026) **(**Fig. 2B**)**. In addition, the 18 patients in the low-immune score group also presented a better survival probability than the 159 patients in the high-immune score group (*P* = 0.019) **(**Fig. 2A**)**. Generally, the above results indicated that stromal and immune scores are both significantly related to the clinical outcomes of PAAD patients.

**Table 1:** Clinicopathologic characteristics of patients with PAAD

Characteristics	Whole cohort (*n* = 177)
**Gender**	
Male	97 (54.80%)
Female	80 (45.20%)
**Age**	
< 65	81 (45.76)
≥ 65	96 (54.24%)
**Grade**	
G1–2	125 (70.62%)
G3–4	50 (28.25%)
Unknow	2 (1.13%)
**Histology type**	
Pancreatic ductal adenocarcinoma	145 (81.92%)
Pancreas colloid carcinoma	4 (2.26%)
Pancreas undifferentiated carcinoma	1 (0.56%)
Other subtype	27 (15.26%)
**T stage**	
TI-II	31 (17.51%)
TIII-IV	144 (81.36%)
Unknow	2 (1.13%)
**N stage**	
N0	50 (28.25%)
N1	122 (68.93%)
Unknow	5 (2.82%)
**AJCC Stage**	
I-II	166 (93.79%)
III-IV	9 (5.08%)
Unknow	2 (1.13%)
**Survival status**	
Dead	92 (51.98%)
Alive	85 (48.02%)

### Identification of DEASs in PAAD from the perspective of the TIME

**Fig. 3:**
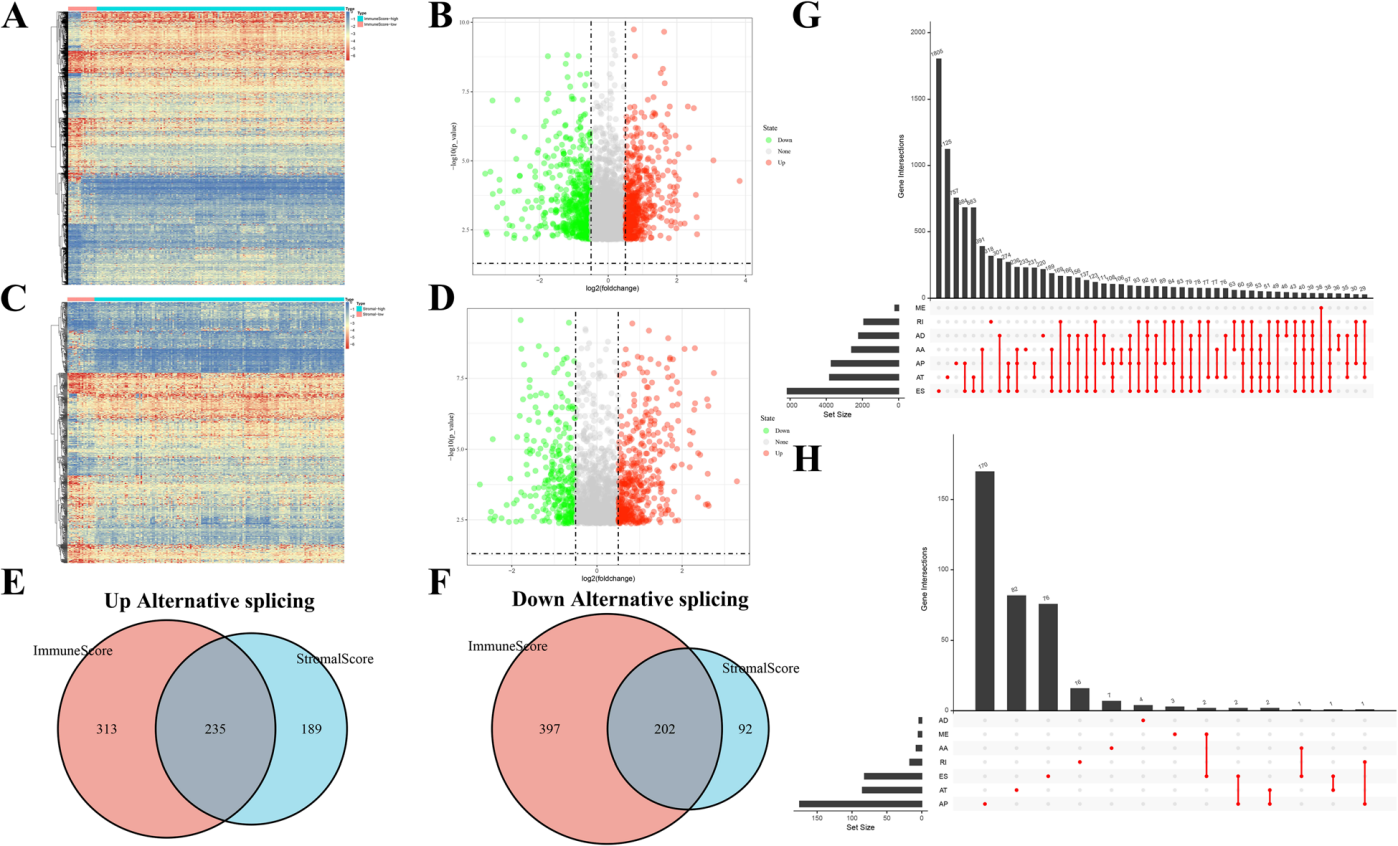
Identification of DEASs in PAAD from the perspective of the TIME. **A** Heatmap of 1147 DEASs between different immune score groups. **B** Volcano plot of 1147 DEASs between different immune score groups; the red points indicate that the log2FC of the DEAS is greater than 0.5, while the green points indicate that the log2FC is lower than − 0.5. **C** Heatmap of 718 DEASs between different stromal score groups. **D** Volcano plot of 718 DEASs between different stromal score groups. **E** Venn diagram of 235 intersecting DEASs in the high-immune score and high-stromal score groups. **F** Venn diagram of 202 intersecting DEASs in the low-immune score and low-stromal score groups. **G** UpSet plot of ASs before screening. **H** UpSet plot of DEASs after screening. DEASs: Differentially expressed alternative splicing events; PAAD: Pancreatic adenocarcinoma; TIME: Tumour immune microenvironment

As displayed in Fig. 2C, seven types of ASs were extracted from TCGA-SpliceSeq to identify potential ASs related to the formation of the TIME. From the perspective of immunity, the differential analysis between the low-immune score group (*n* = 18) and the high-immune score group (*n* = 156) revealed 1147 DEASs **(**Supplementary Table 1**)**, including 548 upregulated DEASs and 599 downregulated DEASs **(**Fig. 3A-B**)**. For stromal cells, 718 corresponding DEASs between the low-stromal score group (*n* = 17) and the high-stromal score group (*n* = 157) were identified (Supplementary Table 2), including 424 upregulated DEASs and 294 downregulated DEASs **(**Fig. 3C-D**)**. Notably, intersecting DEASs, which were interpreted as either upregulated in both the high-immune and high-stromal score groups or downregulated in both the low-immune and low-stromal groups, were identified to be the most relevant genes associated with the TIME and prognosis of PAAD. Thus, 235 commonly upregulated DEASs and 202 commonly downregulated DEASs were mined, as shown in Venn diagrams **(**Fig. 3E-F**)**. To intuitively illustrate the distribution characteristics of ASs and their mutual intersections, two UpSet plots were generated, including one before screening **(**Fig. 3G) and one after screening **(**Fig. 3H**)**. Interestingly, ES events were the most frequent events before screening, and the numbers of AP and AT events associated with TIME formation were the highest. In summary, 437 candidate TIME-related DEASs were significantly associated with the OS of PAAD patients and warrant further research.

### Functional enrichment analysis

**Fig. 4:**
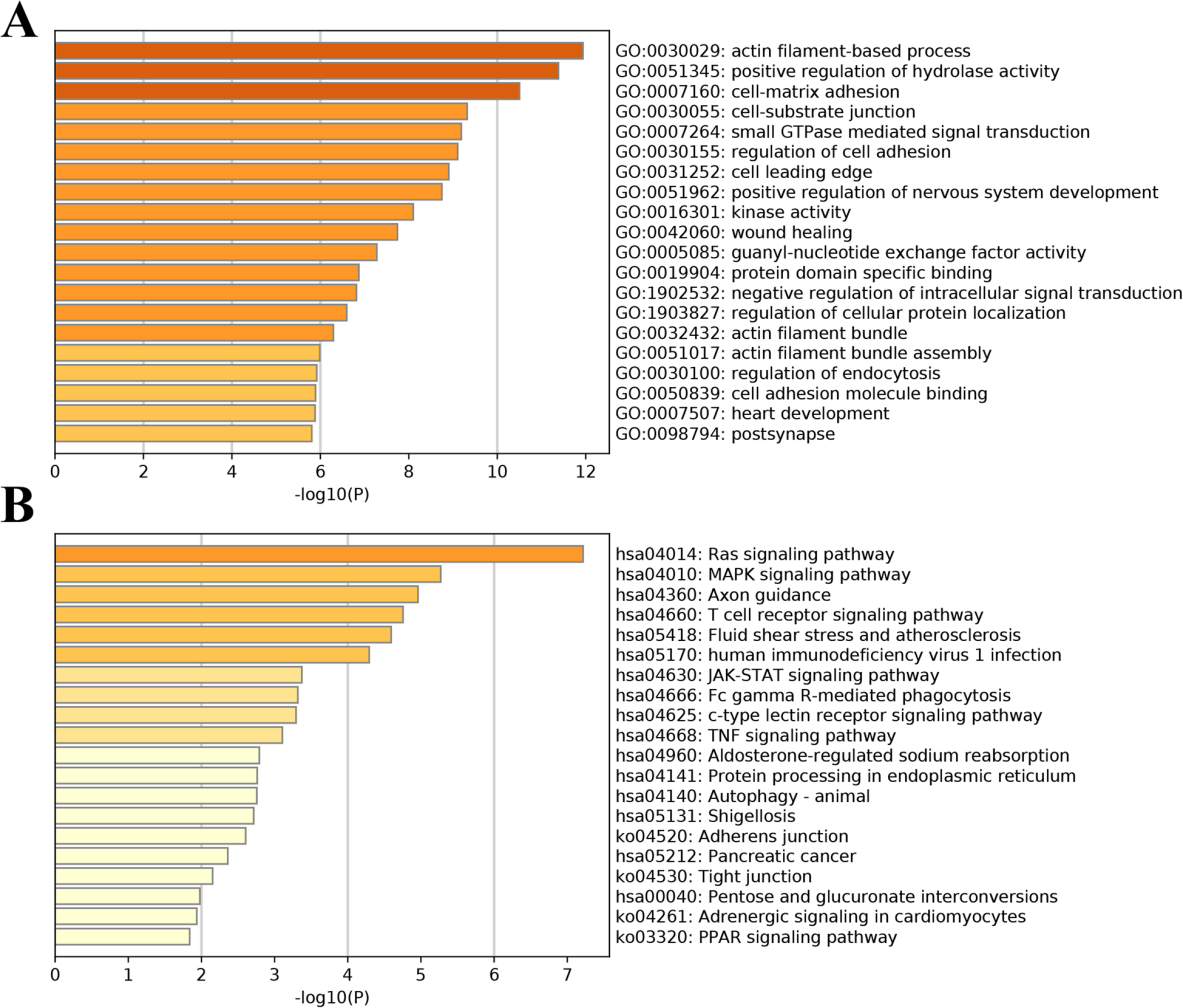
Functional enrichment analysis of 437 candidate DEASs. **A** Bar graph of the 20 most significant terms from GO functional annotation. **B** Bar graph of the top 20 results from the KEGG pathway analysis

The results of GO and KEGG annotation analyses are displayed in Fig. 4A-B. We found that the top 20 results of the GO analysis included actin filament base process, cell-matrix adhesion, cell-substrate junction, regulation of cell adhesion, protein domain-specific binding, protein domain-specific binding, guanyl-nucleotide exchange factor activity, small GTPase-mediated signal transduction, positive regulation of hydrolase activity, regulation of cellular protein localization, and cell adhesion molecule binding **(**Fig. 4A**)**, the dysregulation of which are pivotal factors in carcinogenesis and progression. In addition, KEGG pathway analysis also revealed some tumour-related and immune-related pathways, including the Ras signalling pathway, the T cell receptor signalling pathway, human immunodeficiency virus 1 infection, the JAK-STAT signalling pathway, the MAPK signalling pathway, autophagy, the PPAR signalling pathway, and the TNF signalling pathway **(**Fig. 4B**)**. The results indicated that TIME-related DEASs may serve as important intermediaries in PAAD tumorigenesis, and stromal and immune cell dysfunction can indirectly or directly affect the biological activity of PAAD cells, including proliferation, apoptosis inhibition, and immune tolerance.

### Construction of the TIME-related prognostic signature based on DEASs

**Fig. 5:**
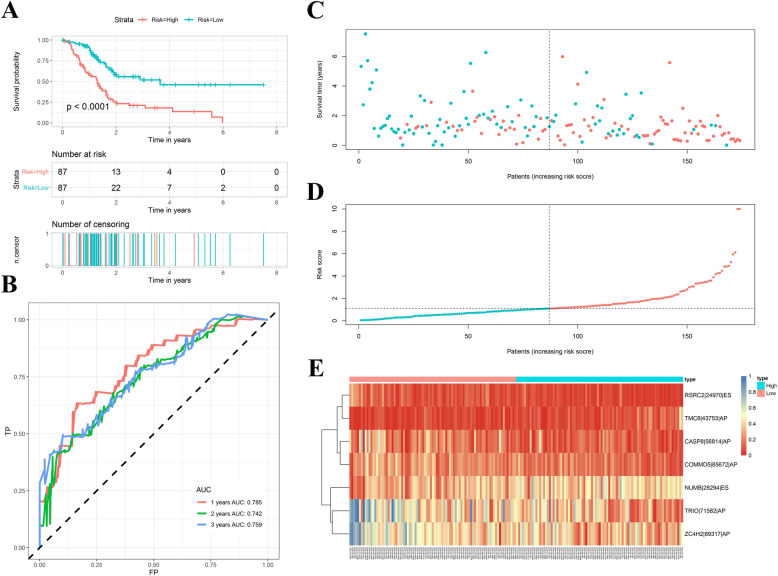
Development of a TIME-related prognostic signature based on DEASs. **A** Kaplan–Meier survival curves showing the differences in OS between patients with different risk levels. **B** Time-dependent ROC curves of the prognostic signature at 1–3 years. **C** RiskScore distribution of patients between different risk groups. **D** Survival status scatter plots for patients between different risk groups. **(E)** Heatmap of seven DEASs between the high- and low-risk groups

Early diagnosis of cancers and detection of underlying targets remain essential problems in clinical practice, and ASs in the TIME may serve as biomarkers with prognostic value in PAAD. Therefore, we further explored the potential prognostic significance of DEASs identified in the above study. First, among 437 intersecting DEASs, 183 DEASs were determined to be significantly related to the OS of PAAD patients by univariate Cox regression analysis (Supplementary Table 3). Subsequently, we performed LASSO regression analysis (Supplementary Fig. 2), and 16 optimal OS-related DEASs were identified. Ultimately, multivariate Cox analysis was implemented, and seven DEASs were utilized to establish a TIME-related prognostic signature (Supplementary Table 4). Additionally, riskScores were calculated based on the seven DEASs (riskScore = 2.125* NUMB|28,294|ES - 3.606* RSRC2|24,970|ES + 3.509* TMC6|43,753|AP - 2.466* CASP8|56,814|AP - 1.678* TRIO|71,582|AP - 1.706* ZC4H2|89,317|AP -2.743* COMMD5|85,672|AP), and PAAD patients were classified into high- and low-risk groups based on the median value. As shown in Fig. 5A, compared with the patients in the high-risk group (*n* = 87), the patients in the low-risk group (*n* = 87) presented a significantly lower incidence of death and favourable OS. Furthermore, the time-dependent ROC curves demonstrated that the AUCs of the TIME-related prognostic signature for evaluating 1-, 2-, and 3-year OS were 0.785, 0.742, and 0.759, respectively **(**Fig. 5B**)**, indicating that the signature can serve as a precise predictive tool. To visually show differences in riskScores and the expression levels of the seven DEASs between different risk groups, riskScore plots, survival plots, and expression heatmaps **(**Fig. 5C-E**)** were generated. Additionally, considering that histologically different PAAD types arising from different cells may present differing prognoses, we further evaluated the predictive power of the signature in patients with pancreatic ductal adenocarcinoma (PDAC). As shown in Supplementary Fig. 3A-B, the AUCs of PDAC patients for predicting 1-, 2-, and 3-year OS were 0.773, 0.703, and 0.695, respectively, and KM survival curves also revealed that high-risk PDAC patients had a worse prognosis.

### Development of the AS clinical nomogram integrating the TIME-related signature and clinical parameters

**Fig. 6:**
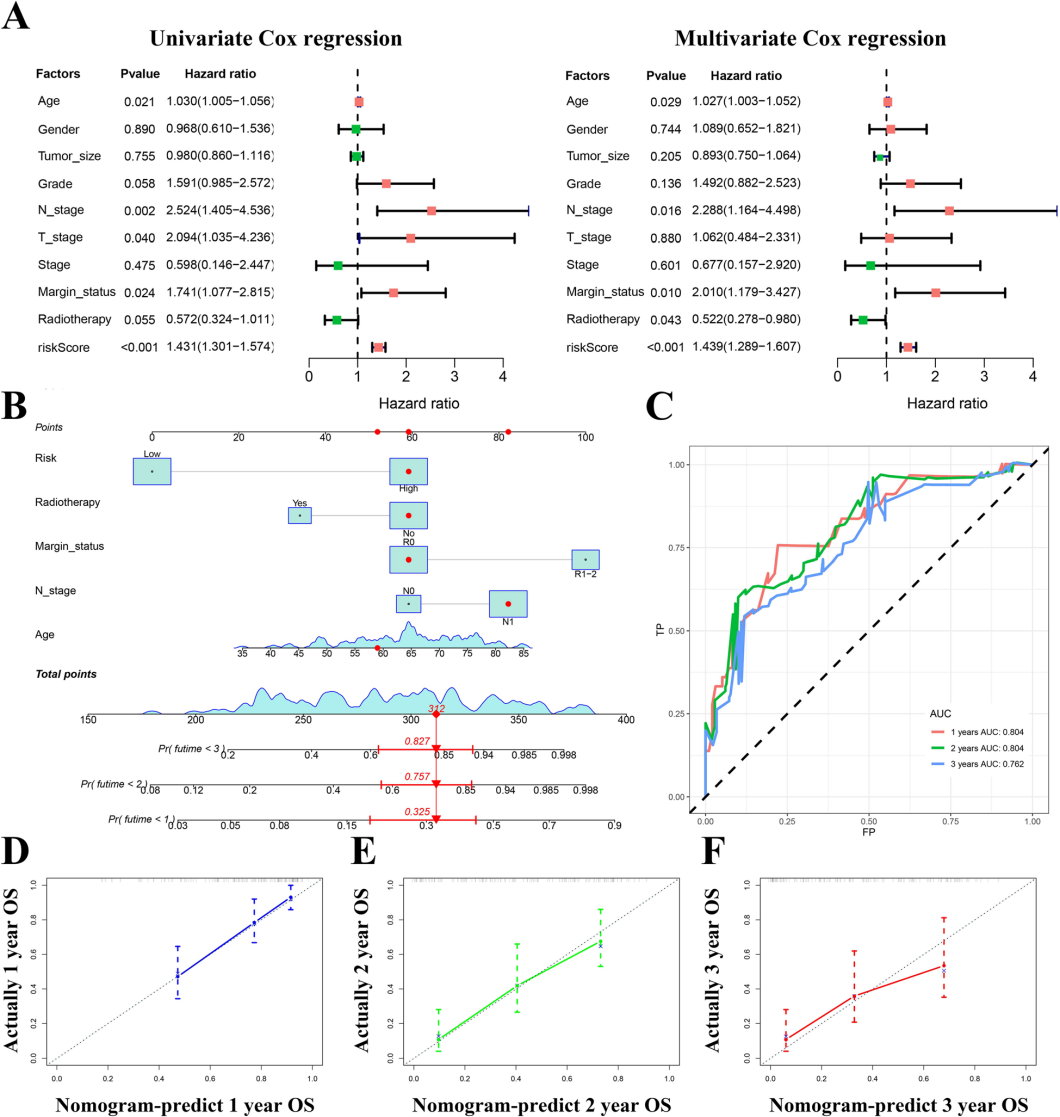
Development of an AS clinical nomogram on the basis of the TIME-related signature and clinical parameters. **A** Univariate and multivariate Cox regression analyses of the TIME-related signature and clinical parameters. **B** A compressive AS clinical nomogram for predicting 1–3 year OS for PAAD patients. **C** Time-dependent ROC curves of the AS clinical nomogram at 1–3 years. **D-F** Calibration curves of the AS clinical nomogram at 1–3 years

To further understand the prognostic significance of the AS signature for clinical application, univariate and multivariate Cox regression analyses were implemented. As displayed in Fig. 6A, the Cox analyses revealed that in addition to the riskScore being one of the independent prognostic parameters for PAAD patients, age, N stage, and margin status were also independent OS-related factors. Subsequently, on the basis of four independent variables, a quantitative AS clinical nomogram was constructed for the risk assessment of survival in newly diagnosed PAAD patients **(**Fig. 6B**)**. The C-index was 0.755 (95%CI = 0.694 ~ 0.816). Similarly, the ROC curves showed that the AUCs of the nomogram were 0.804, 0.804 and 0.762 at 1, 2, and 3 years **(**Fig. 6C**)**, respectively, which were significantly higher than those for the single TIME-related prognostic signature. Moreover, the calibration curves for the 1- to 3-year OS probabilities for PAAD patients also indicated good consistency between the nomogram-predicted outcome and the actual result **(**Fig. 6D-F**)**. Similarly, we also tested the predictive power of the nomogram in PDAC patients (Supplementary Fig. 3C-D). Compared with the low-risk group patients, PDAC patients in the high-risk group presented an extremely poor prognosis, and the AUCs were 0.801, 0.780, and 0.707 at 1, 2, and 3 years, respectively. These results suggested that the comprehensive AS clinical nomogram exhibited a stable and robust ability to evaluate the prognosis of PAAD patients.

### Pathway and immune signature enrichment analyses and GSEA

**Fig. 7:**
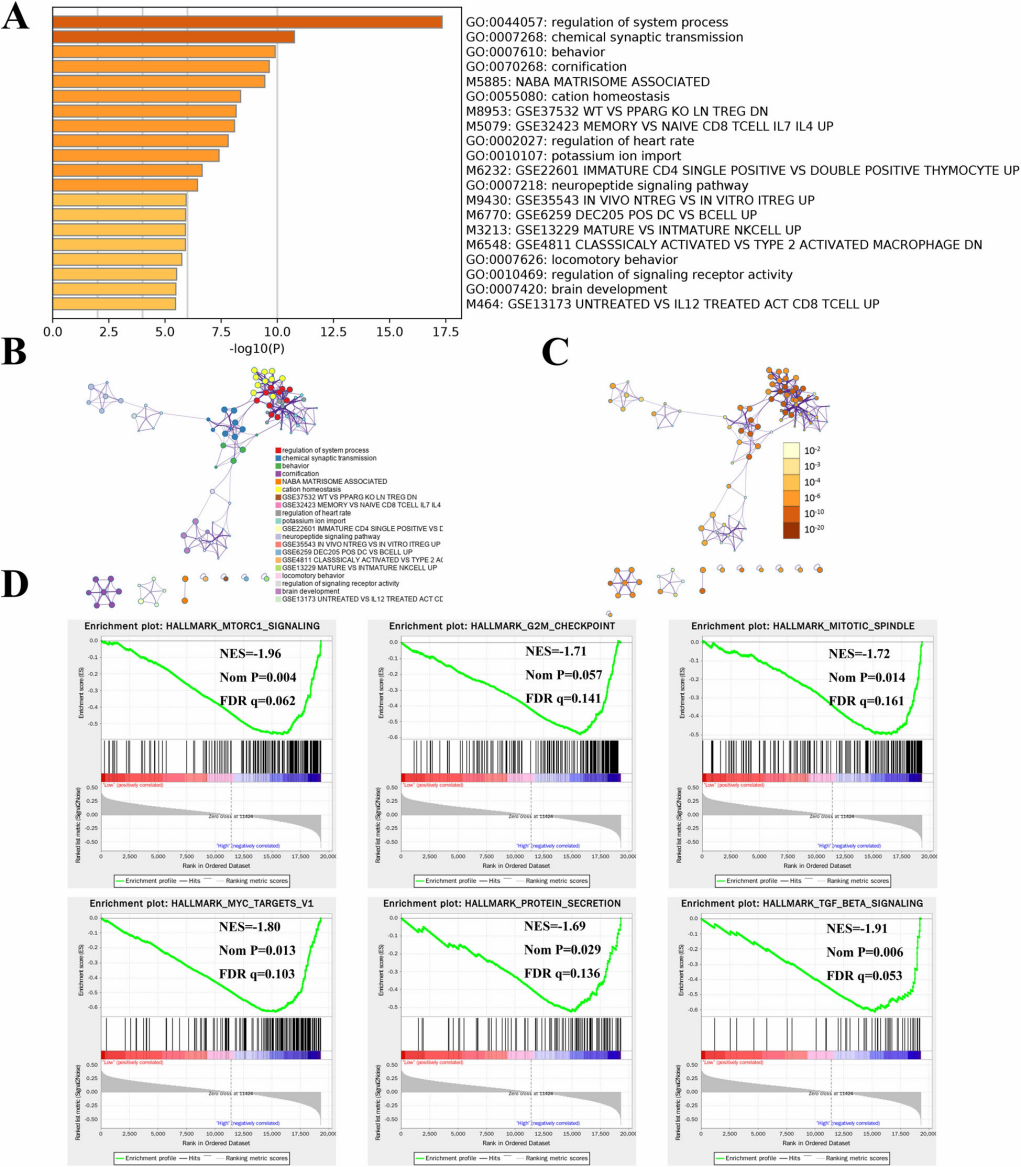
Pathway and immune signature enrichment analyses and GSEA. **A** Bar graph of the 20 most significant terms from the GO functional annotation and immune signature analysis. **B-C** Cluster ID and *p* value of the results of enrichment analysis. **D** The top six tumour hallmarks were enriched in the high-risk patients according to the GSEA

To explore the underlying biological activities and immune signatures involved in the heterogeneity between different risk groups, 597 differentially expressed genes (DEGs) were identified (*P* < 0.05 and |log2FC| > 1) (Supplementary Table 5). These DEGs were mainly enriched in “WT vs. PPARG KO LN TREG DN”, “MEMORY vs. NAÏVE CD8 TCELL IL7 IL4 UP”, “IMMATURE CD4 SING POSITIVE vs. DOUBLE POSITIVE THYMOCYTE UP”, “CLASSSICALY ACTIVATED vs. TYPE 2 ACTIVATED MACROPHAGE DN”, “NABA MATRISOME ASSOCIATED”, “IN VIVO NTREG vs. IN VITRO ITREG UP”, “VIVO NTREG vs. IN VITRO ITREG UP”, “DEC205 POS DC vs. BCELL UP”, “MATURE vs. INTMATURE NKCELL UP”, and “UNTREATED vs. IL12 TREATED ACT TCELL UP”, with immune-associated signatures accounting for most results **(**Fig. 7A). Furthermore, GSEA determined that several cancer hallmarks were significantly higher in the high-risk groups, including Mtorc1 signalling, Myc targets v1, mitotic spindle, protein secretion, TGF-beta signalling, and G2M checkpoint **(**Fig. 7D). These data may provide novel insights into the biological activities associated with TIME-related DEASs.

### Evaluation of tumour mutation and immune characteristics between different risk groups

**Fig. 8:**
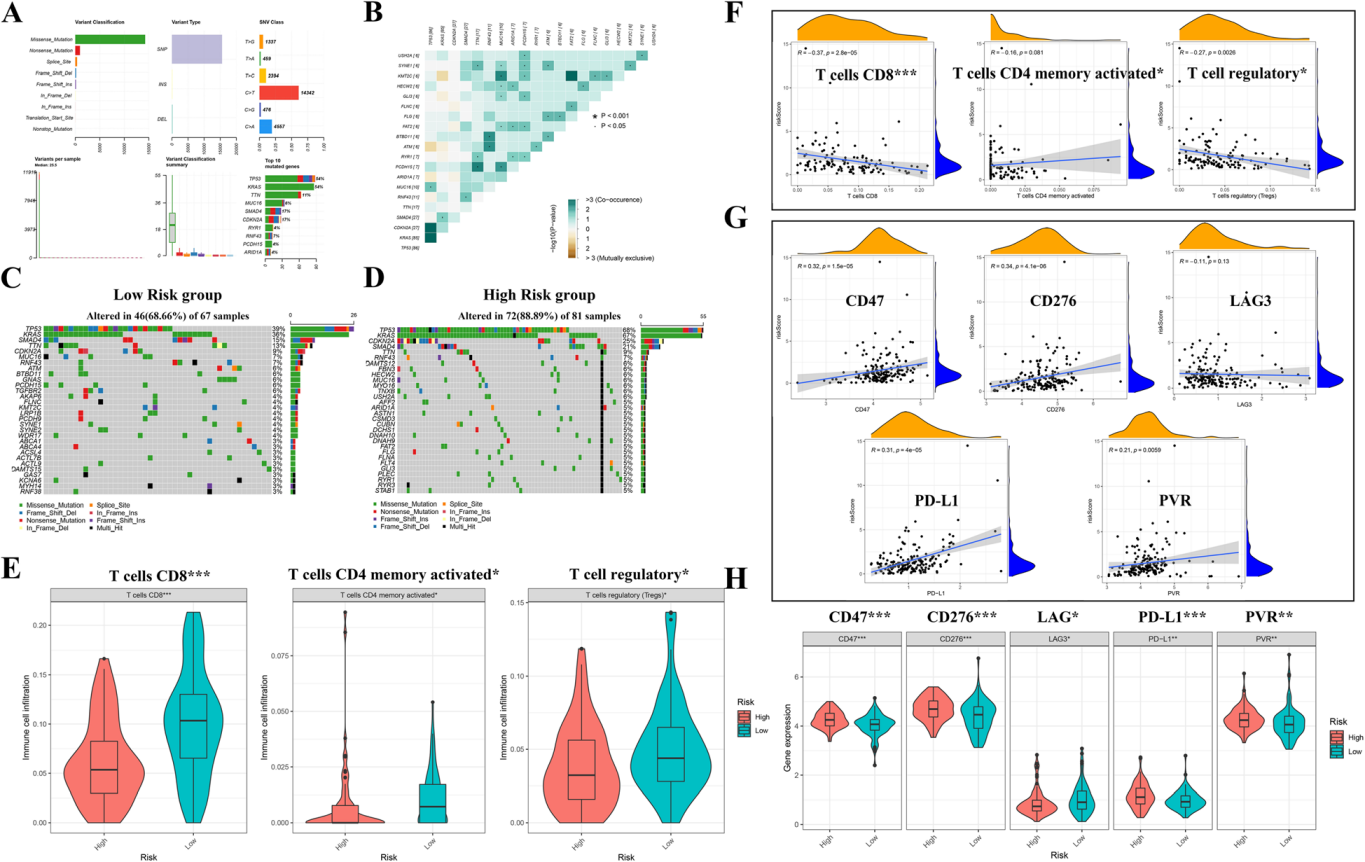
Evaluation of mutation patterns and immune features between different risk groups. **A** Overall view of mutation patterns in PAAD patients. **B** The coexpression patterns of the top 20 mutated genes in PAAD patients. **C-D** Waterfall plot visualizing the top 30 genes that mutated most frequently in the low-risk and high-risk groups. **E** The infiltration levels of CD8 T cells, regulatory T cells, and memory CD4 T cells activated between different risk groups. **F** Correlation analysis between riskScores and CD8 T cells (R = -0.37, *P* < 0.0001), activated CD4 memory T cells (R = -0.16, *P* = 0.081), and regulatory T cells (R = -0.27, *P* = 0.0026). **G** Correlation analysis between riskScores and CD47 (R = 0.32, P < 0.0001), CD276 (R = 0.34, P < 0.0001), LAG3 (R = -0.11, *P* = 0.13), PD-L1 (R = 0.31, P < 0.0001), and PVR (R = 0.21, *P* = 0.0059). **H** The expression of CD47, CD276, LAG, PD-L1, and PVR between the high- and low-risk groups. PAAD: Pancreatic adenocarcinoma

The tumour mutation burden (TMB) has a vital role in tumour occurrence and progression and affects the immunotherapy response and prognosis of PAAD [29]. According to mutation data, 118 PAAD patients had mutated genes, and the top five mutated genes were TP53, KRAS, TTN, MUC16, and SMAD4 **(**Fig. 8A**)**. Additionally, somatic interactions among the top 20 mutated genes were generated **(**Fig. 8B**)**. Then, we compared the most frequent somatic mutations between different risk groups. As displayed in Fig. 8C and D, among the top five mutated genes, the mutation frequencies of TP53 (68% vs. 39%), KRAS (67% vs. 36%), CDKN2A (25% vs. 9%), and SMAD4 (21% vs. 15%) were higher in the high-risk group, indicating that patients in the high-risk group were more suitable for immune checkpoint blockers (ICBs) to achieve a better prognosis.

A comprehensive understanding of different tumour immune phenotypes plays an essential role in predicting immunotherapeutic responsiveness [14]. Thus, we evaluated the infiltration levels of 22 types of immune cells in the TIME and the expression of 15 immune checkpoints between the low- and high-risk groups (Supplementary Figs. 4 and 5). Overall, most immune checkpoints and infiltrating immune cells were not significantly different. However, the infiltration levels of CD8 T cells (*P* < 0.0001), activated CD4 memory T cells (*P* = 0.029), and regulatory T cells (*P* = 0.0187) were higher in the low-risk group **(**Fig. 8E), which indicated that activated T cells in the TIME could inhibit the biological activity and growth of tumour cells, leading to a favourable prognosis. For the five significant immune checkpoints between the two risk groups, the high-risk group had higher expression levels of CD276, PVR, CD47, and PD-L1 and tended to have a positive response to specific ICBs (Fig. 8 H), which was consistent with the mutation pattern results. In addition, the associations between riskScores and the three significant T cell types, including CD8 T cells (R = -0.37, *P* < 0.0001), activated memory CD4 T cells (R = -0.16, *P* = 0.081), and regulatory T cells (R = -0.27, *P* = 0.0026) **(**Fig. 8F**)**, and five immune checkpoint genes, including CD47 (R = 0.32, *P* < 0.0001), CD276 (R = 0.34, *P* < 0.0001), LAG3 (R = -0.11, *P* = 0.13), PD-L1 (R = 0.31, *P* < 0.0001), and PVR (R = 0.21, *P* = 0.0059) **(**Fig. 8G**)**, were analysed. The results revealed that the TIME-related prognostic signature is also promising and effective for recognizing patients’ response to immunotherapy.

### Construction of the AS-SF regulatory network

To investigate whether DEASs are regulated by particular SFs in PAAD, we first identified 48 pairs of interactions between six SFs and 10 DEASs (Supplementary Table 6). A correlation plot was drawn to illustrate concrete correlation coefficients among 48 pairs of interactions between different risk groups (Supplementary Fig. 6A), and the AS-SF regulatory network is visualized in Supplementary Fig. 6B. Interestingly, we found that one SF can regulate different ASs, and some ASs can also be regulated by different SFs, revealing complex cooperative and competitive relationships.

## Discussion

Tumours are a complex mixture of malignant cells, stromal cells, and immune cells that usually have substantial levels of intratumour and intertumour heterogeneity. In addition to these cells, the TIME also includes a combination of tumour-promoting and antitumour signals that can be used to develop effective immunotherapies [30]. However, the failure of immunotherapy to improve the prognosis of PAAD might be explained by both the high molecular heterogeneity of this disease and low immune activation [31]. The immunosuppressive nature of the PAAD TIME is characterized by inhibition of effector T cells or antigen-presenting cells and a strong barrier created by tumour cells to exclude immune cells [32–34]. In recent years, ASs, which are regarded as the mechanisms by which pre-mRNA is edited to acquire mature mRNA, have been found to have significant relationships with TIME formation. Therefore, performing a comprehensive analysis of ASs is a promising strategy for characterization of the TIME and elucidation of the role of ASs in immunotherapy and prognosis prediction. Our study is the first to report that PAAD patients with different abundance levels of stromal cells and immune cells showed significantly different prognostic outcomes. Based on the TIME-related DEASs, a prognostic signature showing independent predictive ability and an AS clinical nomogram were established for precise prognostic predictions. In addition, we determined that the signature built from the perspective of the TIME has good performance in assessing the tumour mutation burden, immune cell infiltration, especially CD8 T cells, the expression levels of five immune checkpoint genes, and the response to immunotherapy.

Tumour-stromal extracellular matrix interactions are critical in PAAD pathophysiology, and more advanced TIME studies are needed to better understand the mechanisms of PAAD [35]. With the aim of elucidating the potential effect of the TIME on PAAD cells, we implemented the ESTIMATE algorithm to infer the proportions of stromal and immune cells and calculated corresponding scores. To explore the role of ASs in the context of the TIME and prognosis of PAAD, 437 TIME-related DEASs were obtained. Functional annotation analysis determined that DEASs were primarily involved in actin filament base processes, cell-matrix adhesion, cell-substrate junctions, regulation of cell adhesion, negative regulation of intracellular signal transduction, and small GTPase-mediated signal transduction, the dysregulation of which may lead to the occurrence and progression of PAAD [36, 37]. Continuous cell-cell and cell-matrix interactions maintain the TIME; thus, fully understanding the latent mechanisms underlying DEASs helps to overcome hurdles in immunotherapeutic strategies. Moreover, the KEGG pathway analysis also implied that TIME-related DEASs may have clinical application potential in PAAD. To achieve constant proliferation, PAAD cells need a continuous RAS signalling pathway and MAPK signalling pathway. Any mutations that inactivate GTPase constitutively activate Ras signalling and induce PAAD progression [38]. The recruited and activated MAPK signalling pathway elements lead to the inflammation, apoptosis, proliferation, and carcinogenesis of pancreatic cells [39]. Increasing evidence has revealed that the infiltration and preferential accumulation of tumour antigen-specific T cells in PAAD are crucial to PAAD cell clearance and long-term remission [40].

In the present study, the clinical significance of DEASs was also explored. A TIME-related prognostic signature based on seven DEASs was constructed and validated to be an independent predictive tool. Some abnormally regulated genes in our prognostic signature participated in tumour initiation and development, including NUMB, RSRC2, TMC6, CASP8, TRIO, and COMMD5. NUMB endocytic adaptor protein (NUMB), a cell fate determinant in asymmetric cell division, is strongly correlated with the development and progression of pancreatic cancer [41]. Wang et al. reported that SRPK2 actively increases cell invasion and migration and chemotherapy resistance in oxaliplatin-treated PAAD cells [42]. RSRC2, a tumour suppressor gene, was first found to inhibit oesophageal cancer cell proliferation and affect survival [43]. Liu et al. found that TRA2A can target RSRC2 AS to confer paclitaxel resistance and promote tumour progression in breast cancer [44]. Imahorn et al. revealed a novel TMC6/8 splice site mutation interlinked with HPV infection and cervical cancer [45]. CASP8 plays a significant role in the apoptosis pathway, and its abnormal expression is associated with tumour cell differentiation, the cancer risk, and prognosis [46]. Interestingly, Zou et al. demonstrated that CASP8 inhibited PD-L1 expression by upregulating A20 expression and that decreased CASP8 was associated with sensitivity to anti-PD-L1/PD-1 immunotherapy [47]. Amplification of TRIO and COMMD5 proteins has also been reported in various types of cancer, suggesting an oncogenic function [48, 49]. Hence, our TIME-related signature incorporating these six DEASs might be helpful for early diagnosis and prevention in clinical practice.

Currently, PAAD is a type of tumour considered unsuitable for immunotherapy, and low response rates have been observed in clinical trials [6]. By resolving the unique classes and subclasses of the TIME existing within individual PAAD patients, the ability to predict and direct immunotherapeutic responsiveness will be improved, and new therapeutic targets will be identified. In this study, we mined seven DEASs from the perspective of the TIME and constructed a prognostic signature to evaluate the response to immunotherapy and clinical outcomes of PAAD patients. High-risk group patients with unsatisfying outcomes presented higher mutation patterns and may respond positively to immunotherapy, which is consistent with the conclusion reached by Tang et al. [50]. Unexpectedly, our signature revealed that high-risk PAAD patients have lower infiltration levels of CD8 T cells, regulatory T cells, and activated memory CD4 T cells. Previous studies determined that a lack of CD8+ T cell infiltration in the TIME was the key to the failure of immune checkpoint blockade as an effective treatment for PAAD [51]. In contrast, the results implied that high-risk patients are not suitable for immunotherapy, which is inconsistent with the recommendation deduced from tumour mutation patterns. To further investigate the molecular mechanisms underlying the TIME of PAAD, we compared the expression levels of 15 immune checkpoint genes between different risk groups and found the answer. High-risk patients with significantly higher expression levels of CD276, PVR, CD47, and PD-L1 may be more sensitive to inhibitors of these four target genes, while low-risk patients with higher expression of LAG3 may respond positively to immunotherapy for this target. However, a more detailed understanding of the cross-talk within the TIME of PAAD and the potential mechanism of resistance to immunotherapy requires rigorously designed single-cell RNA-seq studies. In general, we established a precise prognostic prediction tool to infer tumour progression, enhance prognostic precision, and optimize the immunotherapeutic effect. In addition, intricate ASs are orchestrated by restricted SFs, the dysregulation of which is associated with the onset of cancers [52]. An AS-SF regulatory network was constructed to illustrate the transcriptional mechanism of gene regulation, providing a novel perspective for the study of immunotherapeutic targets and resistance mechanisms.

From the perspective of the TIME, we successfully constructed prognostic DEASs, signatures, an AS clinical nomogram, and regulatory networks related to tumorigenesis, the TIME, and immunotherapy. Nevertheless, the present study has some limitations. First, this was a retrospective study, and all data were extracted from the public TCGA database. Due to the limitation of variable AS data, our predictive models were not validated in other databases. Second, although ESTIMATE/CIBERATE algorithms can infer the infiltration levels of immune and stromal cells based on bulk RNA-sequencing data, our research cannot determine which cell types contribute to abnormal ASs. Finally, this study provided novel strategies for predicting and improving PAAD patients’ response to immunotherapy, but further biological experiments and clinical trials are urgently needed to verify the conclusions.

## Conclusion

In summary, we found that PAAD patients with different abundance levels of stromal cells and immune cells showed significantly different prognostic outcomes and extracted a list of DEASs associated with the TIME through the ESTIMATE algorithm. A robust TIME-related prognostic signature based on seven DEASs was constructed to predict the prognosis of PAAD patients, and the signature was significantly correlated with tumour mutation, TIME diversity, immune checkpoint gene expression, and the response to immunotherapy, which may guide advanced decision-making for personalized precision interventions.

## Supplementary Information

**Additional file 1: Supplementary Fig. 1.** PAAD patients were classified into high/low-stromal/immune score groups using X-tile software.

**Additional file 2: Supplementary Fig. 2.** LASSO regression to select the most significant OS-related DEASs.

**Additional file 3: Supplementary Fig. 3.** Validating the predictive ability of TIME-related signature (A-B) and AS clinical nomogram (C-D) in PDAC patients with ROC and KM survival curves.

**Additional file 4: Supplementary Fig. 4.** The infiltration levels of 22 types of immune cells in the TIME between the low- and high-risk groups.

**Additional file 5: Supplementary Fig. 5.** The expression of 15 immune checkpoint genes between low- and high-risk groups.

**Additional file 6: Supplementary Fig. 6.** Construction of ASs-SFs regulatory network. (A) The correlation between 10 ASs and six SFs in PAAD patients. (B). An ASs-SFs regulatory network.

**Additional file 7: Supplementary Table 1.** 1147 differentially expressed alternative splicing events (Immune score).

**Additional file 8: Supplementary Table 2.** 718 differentially expressed alternative splicing events (Stromal score).

**Additional file 9: Supplementary Table 3.** 183 OS-related differentially expressed alternative splicing events.

**Additional file 10: Supplementary Table 4.** The results of multivariate Cox analysis.

**Additional file 11: Supplementary Table 5.** 597 differentially expressed genes between low- and high-risk groups.

**Additional file 12: Supplementary Table 6.** The results of correlation analysis of 6 SFs and 10 DEASs.

## Data Availability

The datasets generated and/or analysed during the current study are available in the TCGA repository (https://portal.gdc.cancer.gov/) and TCGA SpliceSeq (https://bioinformatics.mdanderson.org/TCGASpliceSeq/).

## Abbreviations

PAAD: Pancreatic adenocarcinoma
AS: Alternative splicing event
TIME: Tumor immune microenvironment
KM: Kaplan–Meier
DEAS: Differentially expressed AS
GSEA: Gene set enrichment analysis
SF: Splicing factor
PSI: Percent spliced in
AA: Alternate acceptor site
ES: Exon skip
RI: Retained intron
AT: Alternate terminator
ME: Mutually exclusive exon
AD: Alternative donor site
AP: Alternate promoter
GO: Gene ontology
KEGG: Kyoto encyclopedia of genes and genomes
ROC: Receiver operating characteristic
OS: Overall survival
PDAC: Pancreatic ductal adenocarcinoma
DEG: Differentially expressed gene
TMB: Tumour mutation burden
ICB: Immune checkpoint blocker
