# Supplementary figures and images for: Identification of tumour immune microenvironment-related alternative splicing events for the prognostication of pancreatic adenocarcinoma
Source: BMC Cancer. 2021 Nov 12;21:1211. doi: 10.1186/s12885-021-08962-7 (PMC8590242; doi:10.1186/s12885-021-08962-7)

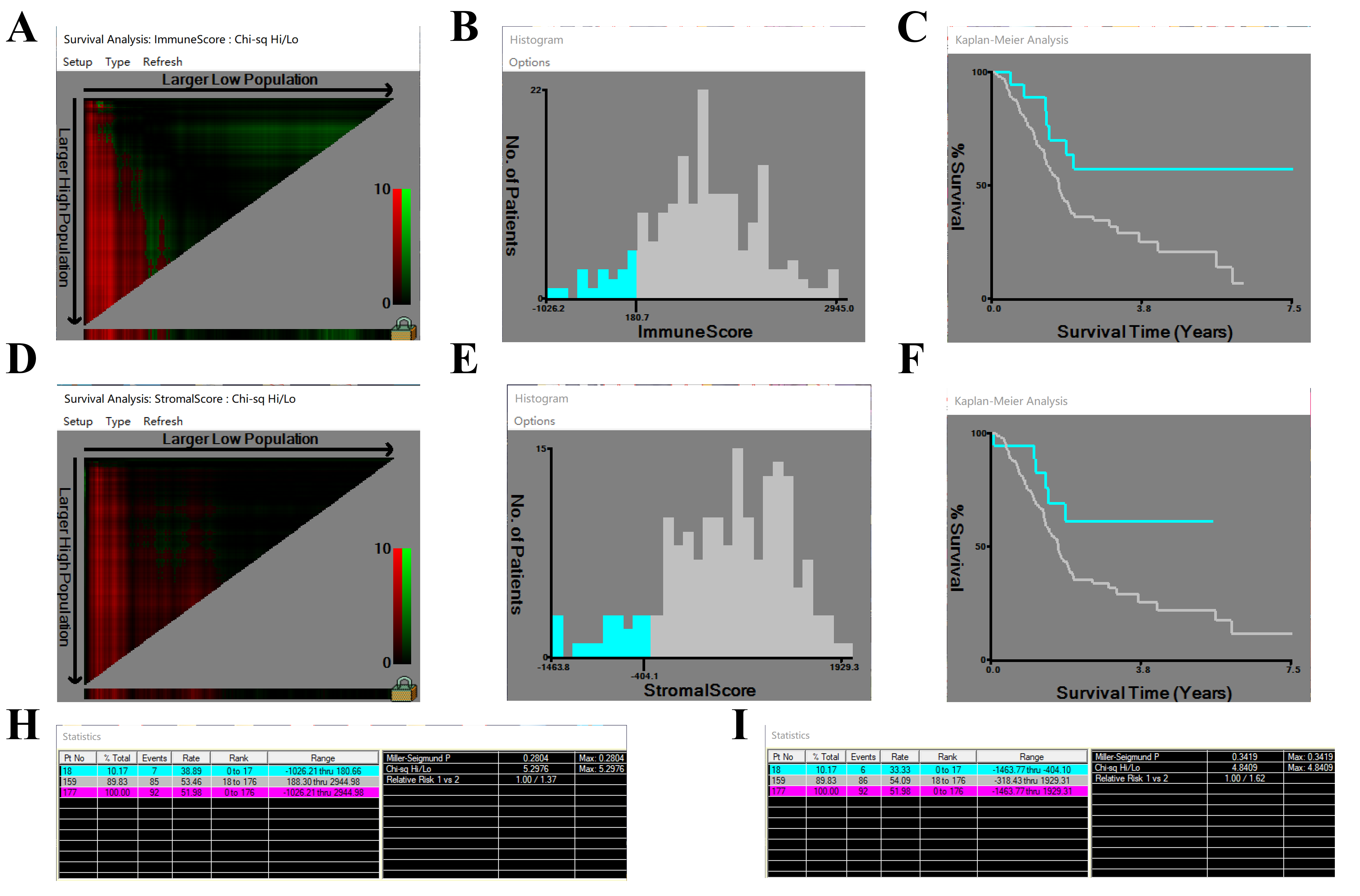

Supplement: Supplementary file 1 — Additional file 1: Supplementary Fig. 1. PAAD patients were classified into high/low-stromal/immune score groups using X-tile software. [file 12885_2021_8962_MOESM1_ESM.tif]

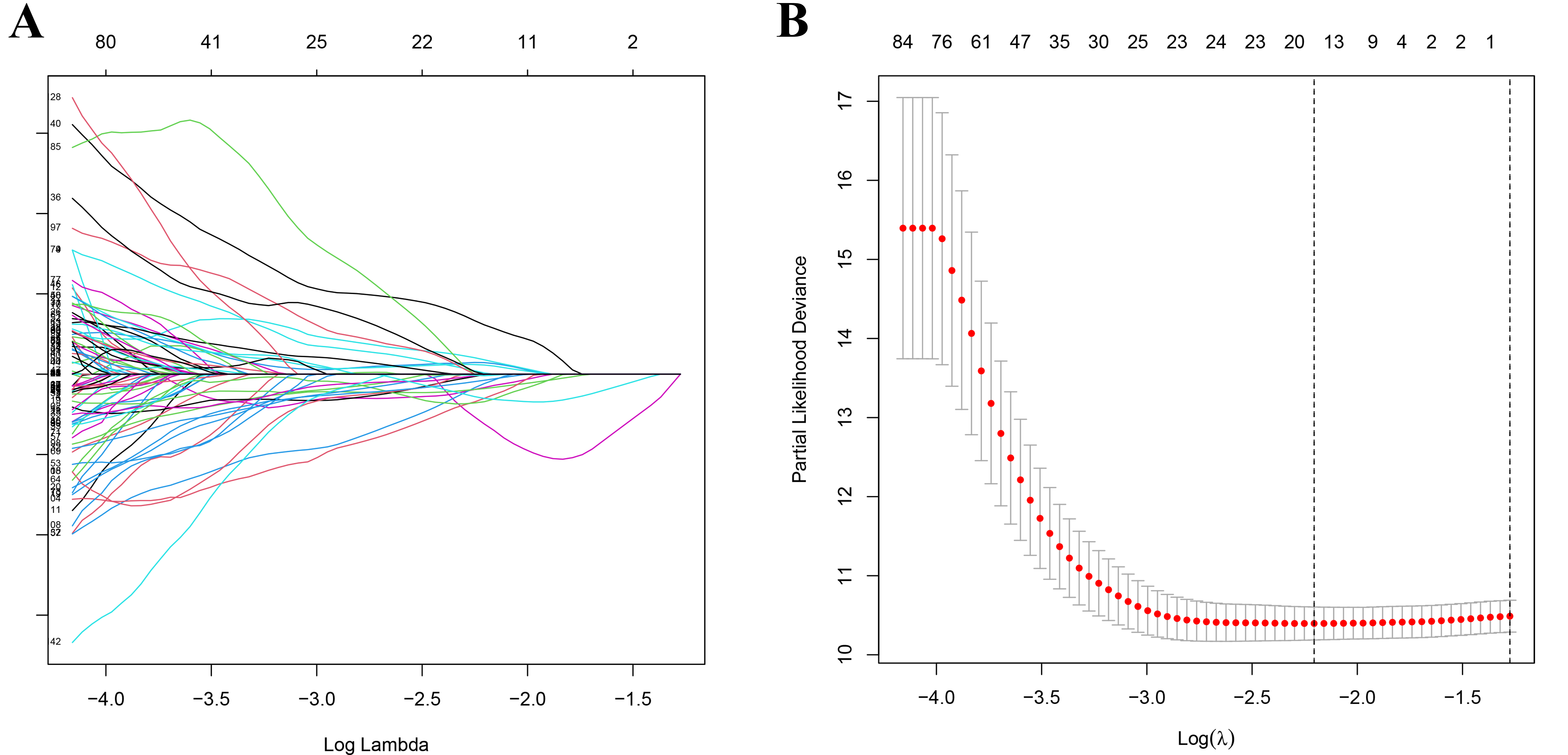

Supplement: Supplementary file 2 — Additional file 2: Supplementary Fig. 2. LASSO regression to select the most significant OS-related DEASs. [file 12885_2021_8962_MOESM2_ESM.tif]

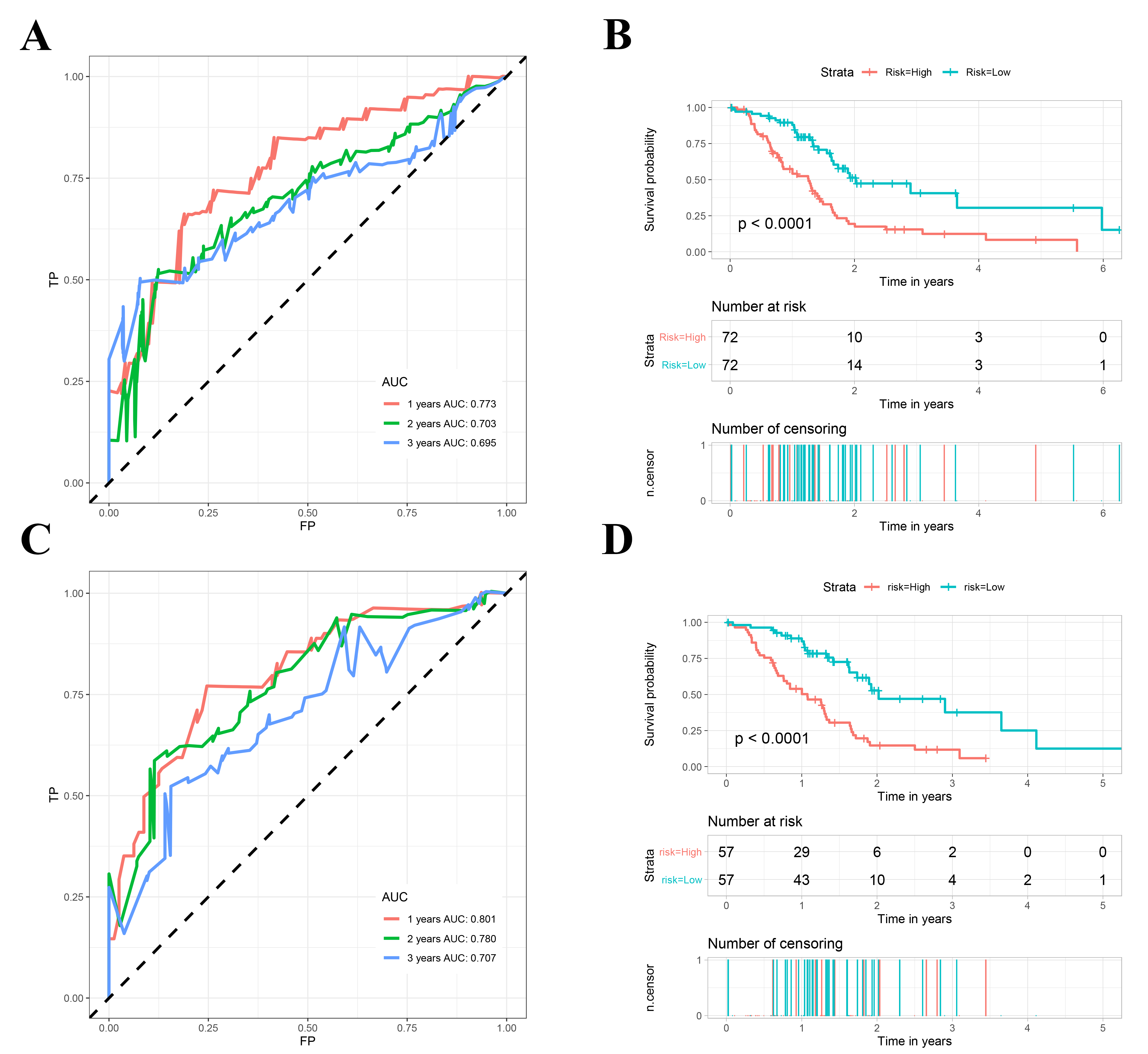

Supplement: Supplementary file 3 — Additional file 3: Supplementary Fig. 3. Validating the predictive ability of TIME-related signature (A-B) and AS clinical nomogram (C-D) in PDAC patients with ROC and KM survival curves. [file 12885_2021_8962_MOESM3_ESM.tif]

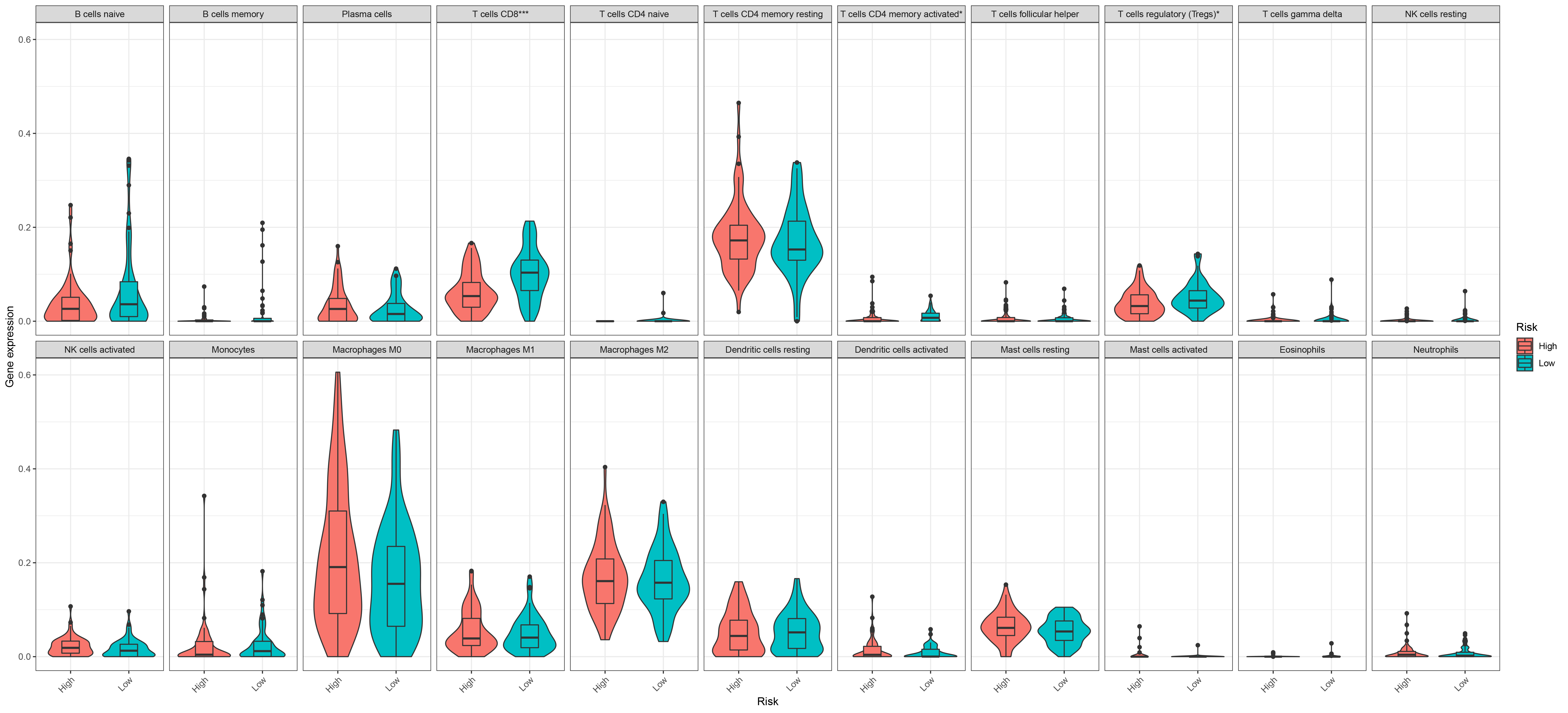

Supplement: Supplementary file 4 — Additional file 4: Supplementary Fig. 4. The infiltration levels of 22 types of immune cells in the TIME between the low- and high-risk groups. [file 12885_2021_8962_MOESM4_ESM.tif]

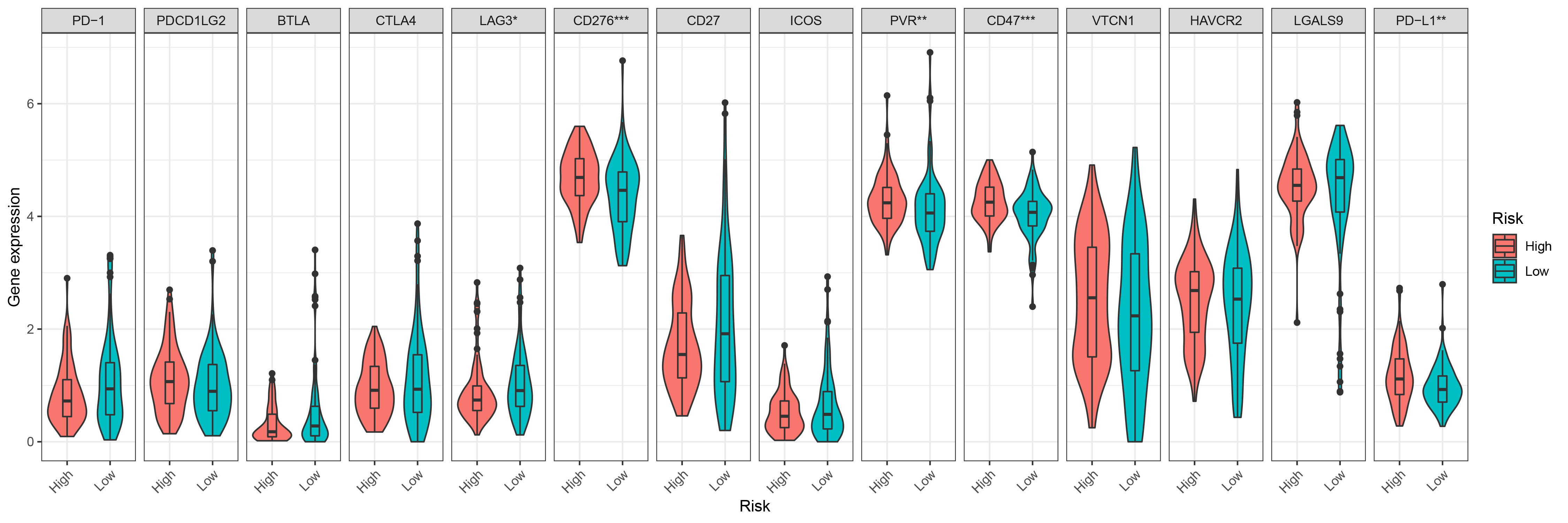

Supplement: Supplementary file 5 — Additional file 5: Supplementary Fig. 5. The expression of 15 immune checkpoint genes between low- and high-risk groups. [file 12885_2021_8962_MOESM5_ESM.tif]
